# Clinical characteristics of Lewy body dementia in Chinese memory clinics

**DOI:** 10.1186/s12883-021-02169-w

**Published:** 2021-03-31

**Authors:** Jinghuan Gan, Shuai Liu, Xiaodan Wang, Zhihong Shi, Lu Shen, Xudong Li, Qihao Guo, Junliang Yuan, Nan Zhang, Yong You, Yang Lv, Dongming Zheng, Yong Ji, China Lewy Body Disease Collaborative Alliance, Shuai Liu, Xiaodan Wang, Zhihong Shi, Lu Shen, Xudong Li, Qihao Guo, Junliang Yuan, Nan Zhang, Yong You, Yang Lv, Dongming Zheng, Yong Ji

**Affiliations:** 1 Department of neurology, Beijing Tiantan Hospital, Capital Medical University, China National Clinical Research Center for Neurological Diseases, Beijing, China; 2 Department of Neurology, Tianjin Huanhu Hospital, Tianjin Key Laboratory of Cerebrovascular and of Neurodegenerative Diseases, Tianjin Dementia Institute, Tianjin, China; 3 Department of Neurology, Xiangya Hospital, Central South University, Changsha, China; 4 Department of Gerontology, Shanghai Jiao Tong University Affiliated Sixth People’s Hospital, Shanghai, China; 5 Peking University Sixth Hospital, Peking University Institute of Mental Health, NHC Key Laboratory of Mental Health (Peking University), National Clinical Research Center for Mental Disorders (Peking University Sixth Hospital), Beijing, China; 6 Department of Neurology, Tianjin Medical University General Hospital, Tianjin, China; 7 Department of Neurology, Second Affiliated Hospital of Hainan Medical University, Haikou, China; 8 Department of Geriatrics, the First Affiliated Hospital of Chongqing Medical University, Chongqing, China; 9 Department of Neurology, Shengjing Hospital Affiliated to China Medical University, Shenyang, China

**Keywords:** Lewy body dementia, Dementia with Lewy bodies, Parkinson’s disease dementia, Prevalence, Gender, Molecular image

## Abstract

**Background:**

Lewy body dementia (LBD), consisting of dementia with Lewy bodies (DLB) and Parkinson’s disease dementia (PDD), is the second most common type of neurodegenerative dementia in older people. The current study aimed to investigate the clinical characteristics of LBD in Chinese memory clinics.

**Methods:**

A total of 8405 dementia medical records were reviewed, revealing 455 patients with LBD. Demographic data, neuropsychological scores, and the scale for Medial Temporal lobe Atrophy (MTA) were then analyzed from nine memory clinics in the China Lewy Body Disease Collaborative Alliance.

**Results:**

The clinical proportion of LBD among the subjects and among all dementia types was 5.4% (4.9–5.9%) and 7.3% (6.7–8.0%), respectively, with a mean onset age of 68.6 ± 8.4 years. Patients with DLB comprised 5.6% (*n* = 348, age of onset 69.1 ± 8.3), while PDD comprised 1.7% (*n* = 107, age of onset 66.7 ± 8.8) of all dementia cases. There were slightly more males than females with DLB (*n* = 177, 50.9%) and PDD (*n* = 62, 57.9%). Patients with DLB had a poorer performance compared to those with PDD on the MMSE (16.8 ± 7.1 vs. 19.5 ± 5.7, *p* = 0.001), the MoCA (11.4 ± 6.6 vs. 14.0 ± 5.8, *p*<0.001), the CDR (1.8 ± 0.7 vs. 1.6 ± 0.7, *p* = 0.002), and the MTA (1.8 ± 0.7 vs. 1.2 ± 0.6, *p* = 0.002). Diagnostic differences for LBD exist among the centers; their reported proportions of those with DLB ranged from 0.7 to 11.4 and those with PDD ranged from 0.0 to 2.9%.

**Conclusions:**

Variations of diagnoses exists in different regions and the clinical proportion of LBD is likely to be underestimated in China and other regions.

## Background

Lewy body dementia (LBD), which includes dementia with Lewy bodies (DLB) and Parkinson’s disease dementia (PDD), is the second most common neurodegenerative dementia, following Alzheimer’s disease (AD), among older people [1]. DLB and PDD, conceptualized as spectrum disorders, are associated with an abnormal accumulation of α-synuclein and have clinical and pathological overlap, with variations in the temporal onset of motor and cognitive symptoms [2]. The prevalence of LBD varies between 15 and 25% among autopsy series diagnoses [3], but clinically is much lower. As previous studies have shown, less than half of the cases could be correctly identified at routine clinical visits [4]. The update of DLB diagnostic criteria suggests using validated and referable biomarkers, however, a crucial lack of accordance complicates the prevalence estimates of DLB.

Patients with DLB comprise 0 to 30.5% of the cases of elderly individuals with dementia in clinical studies [5], while PDD is reported to comprise 3.6–30% [6–8], with estimates of over 80% in PD patients [9]. The DLB prevalence among various population-based studies ranged from 0 to 26% of all dementia cases, consistent with a population-based prevalence (approximately 10%) in rural island town in east Asia [10, 11]. The variation between individual studies’ clinical prevalence has confused clinicians, as it is unclear if these studies represent true differences in LBD prevalence among different regions or countries. Previous studies have emphasized the importance of the diagnosis of LBD, as well as the importance in distinguishing between DLB and PDD, however, the clinical prevalence of DLB and PDD still remains unclear.

In the current study, the diagnoses of patients from nine memory clinics were analyzed and the basic clinical characteristics in patients with DLB to PDD were compared. The current study aimed to understand the clinical prevalence of DLB and PDD in China, to observe gender differences between DLB and PDD samples, and to improve awareness for clinicians.

## Methods

### Participants

**Fig. 1:**
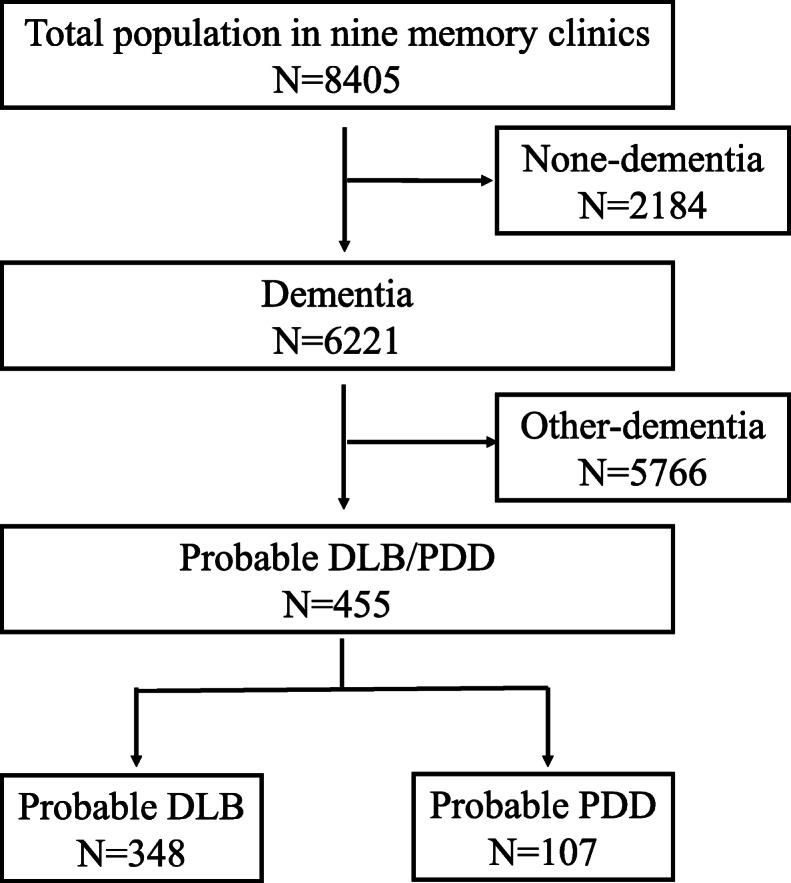
Flowchart of this study. DLB indicates dementia with Lewy bodies; PDD, Parkinson’s disease dementia

A total of 8405 cases were recorded from nine participating hospital memory clinics in China, of which 348 DLB and 107 PDD patients were included (12 patients were diagnosed with unclassifiable dementia) (shown in Fig. 1). The final diagnoses were confirmed by two experienced neurologists, following a case review according to the protocol. Any patients with a questionable diagnosis were excluded from the study and further examination was recommended. Each memory clinic was required to have been open for at least 5 years and to have examined a total of 300 patients with cognitive impairment (CI) from January 1, 2013 to December 31, 2018, with those patients attending routine clinic visits. The nine memory clinics are all tertiary hospitals, residing in the Liaoning (*n* = 1), Beijing (*n* = 2), Tianjin (*n* = 2), Shanghai (*n* = 1), Hunan (*n* = 1), Hainan (*n* = 1), and Chongqing (*n* = 1) provinces. All centers were from the China Lewy Body Disease Collaborative Alliance and showed interest in LBD research.

Demographic data (including gender, age at visit, age at memory decline onset, education), final medical diagnoses, date(s) of consultation, neuroimaging examination [Magnetic Resonance Imaging (MRI) and/or Computed tomography (CT) scan if necessary, 11C-PIB positron emission tomography computed tomography (PET) or 18F-AV45 PET, 18F-FDG PET, dopamine transporter (DAT), and single photon emission computed tomography (SPECT) if available] and neuropsychological scores [Mini Mental State Examination (MMSE) [12], Montreal Cognitive Assessment (MoCA) [13], Activities of daily living (ADL) [14]] for all patients were assessed by reviewing the medical records of the nine units. The Clinical Dementia Rating (CDR) [15] was used to rate the severity of dementia and the scale for Medial Temporal lobe Atrophy (MTA) [16] was used to determine the visual regional brain atrophy for each patient. In the current study, MTA scores were conducted in 131 DLB patients (61 males, 70 females, 1.8 ± 0.7) and 15 PDD patients (10 males, 5 females, 1.2 ± 0.6).

### Diagnostic criteria

Dementia patients were diagnosed according to the criteria for primary degenerative dementia in the fourth edition of the Diagnostic and Statistical Manual of Mental Disorders (DSM-IV) [17]. DLB patients were diagnosed according to the revised consensus criteria for probable DLB, developed in the third report of the DLB consortium [18]. A probable DLB diagnosis can be made with only one core symptom together with one or more suggestive features, such as rapid eye movement sleep behavior disorder (RBD), neuroleptic hypersensitivity, and low dopamine transporter uptake in the basal ganglia, as demonstrated by a SPECT or PET-CT. PDD patients were diagnosed according to the clinical criteria for probable PDD, developed by the Movement Disorder Society (MDS) in 2007 [19]. International consensus suggests that DLB should be diagnosed when cognitive impairment precedes parkinsonism or begins within a year of parkinsonism and PDD should be diagnosed when parkinsonism precedes cognitive impairment by more than 1 year. Therefore, all LBDs (containing DLB and PDD) mentioned in the study were from a probable diagnosis.

### Symptom evaluations

The Mayo Fluctuations Composite Scale was used to confirm the presence of cognitive fluctuations, with three or more “yes” responses required for structured questions from caregivers [20]. Visual hallucinations, specifically formed and detailed visual hallucinations and illusions, that were complained about by the patient and/or caregiver were determined by confirmation and quantification according to the hallucinations item of the NPI [21], while delusions and depression from Parkinsonism were diagnosed by the motor section (Part III) of the Movement Disorders Society Unified Parkinson’s Disease Rating Scale (MDS-UPDRS) [22]. RBD was confirmed by caregivers who mentioned five or more behaviors that are mentioned in the RBD screening questionnaire (RBD-SQ) [23] or someone who was diagnosed using an overnight video polysomnography [24]. Other supportive features such as syncope, falls, orthostatic hypotension, constipation, and hyposmia, were identified through a detailed investigation and assessment and established clinical questionnaires.

### Imaging evaluations

MRI images were obtained using a 3.0 Tesla General Electric scanner or 3.0 T SIEMENS Tim Trio MRI scanner. T1-weighted coronal images were obtained using a 3D spoiled gradient-recalled-echo-inversion recovery prepared sequence (1-mm slice thickness). All images from the scanner were reconstructed to a size of 256 × 256, with an isotropic resolution of 1 × 1 × 1 mm. The visual rates of MTA were quantified and scored blindly by two neurologists using the standardized measures [16], which ranged from 0 to 4. The time interval between the MRI and PET-CT was no longer than 2 weeks.

Either 18F-FDG PET, 11C-PIB PET, or 18F-AV45 PET imaging was conducted by a GE Discovery LS PET/CT scanner or Siemens Biograph mCT Flow PET/CT scanner in 3D scanning mode.

Subjects received an intravenous injection of 240–333 MBq 18F-FDG and a 10 min static PET scan was conducted 40 min later. A voxel-based statistical analysis was performed on the 18F-FDG PET images using Statistical Parametric Mapping (SPM) 8 and Matlab 2010b for Windows. Regions that reached an uncorrected *p*-value < 0.001 were considered statistically significant. Hypometabolism in the lateral occipital cortex and/or relative preservation of the mid or posterior cingulate gyrus (cingulate island sign) indicated a diagnosis of LBD.

Pittsburgh compound-B (PIB) was bolus injected into an antecubital vein at a mean dose of 370–555 MBq. Images from a 90-min dynamic 11C-PIB PET scan were acquired. The 11C-PIB uptake in each cortical region was then calculated, resulting in the selection of a reference cerebellar cortex. 11C-PIB integral images were co-registered to each subject’s T1-weighted MR images. An MRI-based automated region of interest technique was used to sample each subject’s PIB images. Imaging data at 40- to 60-min post-injection were used for the analysis of PIB uptake using parametric images of the PIB standard uptake value ratios (SUVRs). A positive PIB diagnosis was based on both the visual interpretations of elevated binding in the neocortex and the semi-quantitative assessment.

A 20-min 18F-AV45 PET scan was acquired at 50 min post-injection of 248 ± 58 MBq. This data was reconstructed using an ordered subset expectation maximization algorithm with weighted attenuation. Images were smoothed using a 5-mm Gaussian kernel with scatter correction and were evaluated prior to any analysis of patient motion and the adequacy of statistical counts. SUVRs were calculated using the cerebellar gray matter reference region to normalize mean activity from 50- to 70-min. Patients were diagnosed as AV45-positive based on both the visual interpretations of elevated binding in the neocortex and the semi-quantitative assessment.

Given the lack of the other imaging records in this research, neither SPECT nor other imaging evaluations were considered.

Detailed written informed consent was obtained from all subjects and their relatives. The current study was approved by the Ethics Committees of the Tianjin Huanhu Hospital (2011–1). The procedures were performed in accordance with the ethical standards of the Committee on Human Experimentation.

### Statistical analyses

For the statistical analyses, the IBM Statistical Package for the Social Sciences (SPSS) for Windows (version 22.0; IBM Corporation, Armonk, NY) was used. Descriptive analyses were conducted using percent and frequency for qualitative variables and mean with SD for quantitative variables. For comparisons of two independent groups (DLB and PDD), a Student’s t-test was used for normally distributed data and a Mann-Whitney U test was used for nonparametric data. Qualitative variables were assessed using a chi-squared test. A *p*-value of less than 0.05 was considered significant. Adjusted odds ratios (OR) are presented with 95% confidence interval (95% CI). All tests were performed bilaterally.

## Results

### Demographic and clinical characteristics of patients

The researchers reviewed the case notes of 8405 individual patients from nine memory clinics in seven provinces in China, of whom 6221 (74.0%) had a dementia diagnosis (shown in Fig. 1). LBD patients comprised 7.3% (*n* = 455, 95% CI: 6.7, 8.0%) of all dementia cases, and 5.4% (95% CI: 4.9, 5.9) of all samples. The mean age of LBD patients at their first visit was 71.3 (8.8) and the mean age at onset was 68.6 (8.4), with no significant sex-related differences (shown in Table 1). The mean years of education was 10.2 (4.6), with significant sex-related differences (female mean was 9.3 years, male mean was 11.1 years, *p* = 0.001). Among the patients with LBD, men had a significantly higher mean total MoCA score than women (13.0 vs. 11.0, *p* = 0.002), however, there were no significant sex-related differences in the MMSE or ADL scores.

**Table 1:** Demographic: comparison of gender for LBD patients

	All	Male	Female	***p***-value*
**Number of patients, n (%)**	455 (100.0)	239 (52.5)	216 (47.5)	None
**Demographic**				
Age at visit, mean (SD), years	71.3 ± 8.8	71.8 ± 8.3	70.7 ± 8.8	None
Age at onset, mean (SD), years	68.6 ± 8.4	69.2 ± 8.1	68.0 ± 8.8	None
Education, mean (SD), years	10.2 ± 4.6	11.1 ± 4.1	9.3 ± 4.9	0.001
**Diagnosis, n (%)**				
DLB	348 (100.0)	177 (50.9)	171 (49.1)	None
PDD	107 (100.0)	62 (57.9)	45 (42.1)	None
**Clinical Evaluation, mean (SD)**				
MMSE	17.5 ± 6.9	17.9 ± 7.1	17.0 ± 6.6	None
MoCA	12.0 ± 6.5	13.0 ± 6.6	11.0 ± 6.3	0.002
ADL	36.7 ± 15.2	36.1 ± 15.3	37.2 ± 15.2	None

*SD* standard deviation, *DLB* Dementia with Lewy bodies, *PDD* Parkinson’s disease dementia, *MMSE* Mini Mental State Examination, *MoCA* Montreal Cognitive Assessment, *ADL* Activities of daily living

*p-value for male vs. female

**Fig. 2:**
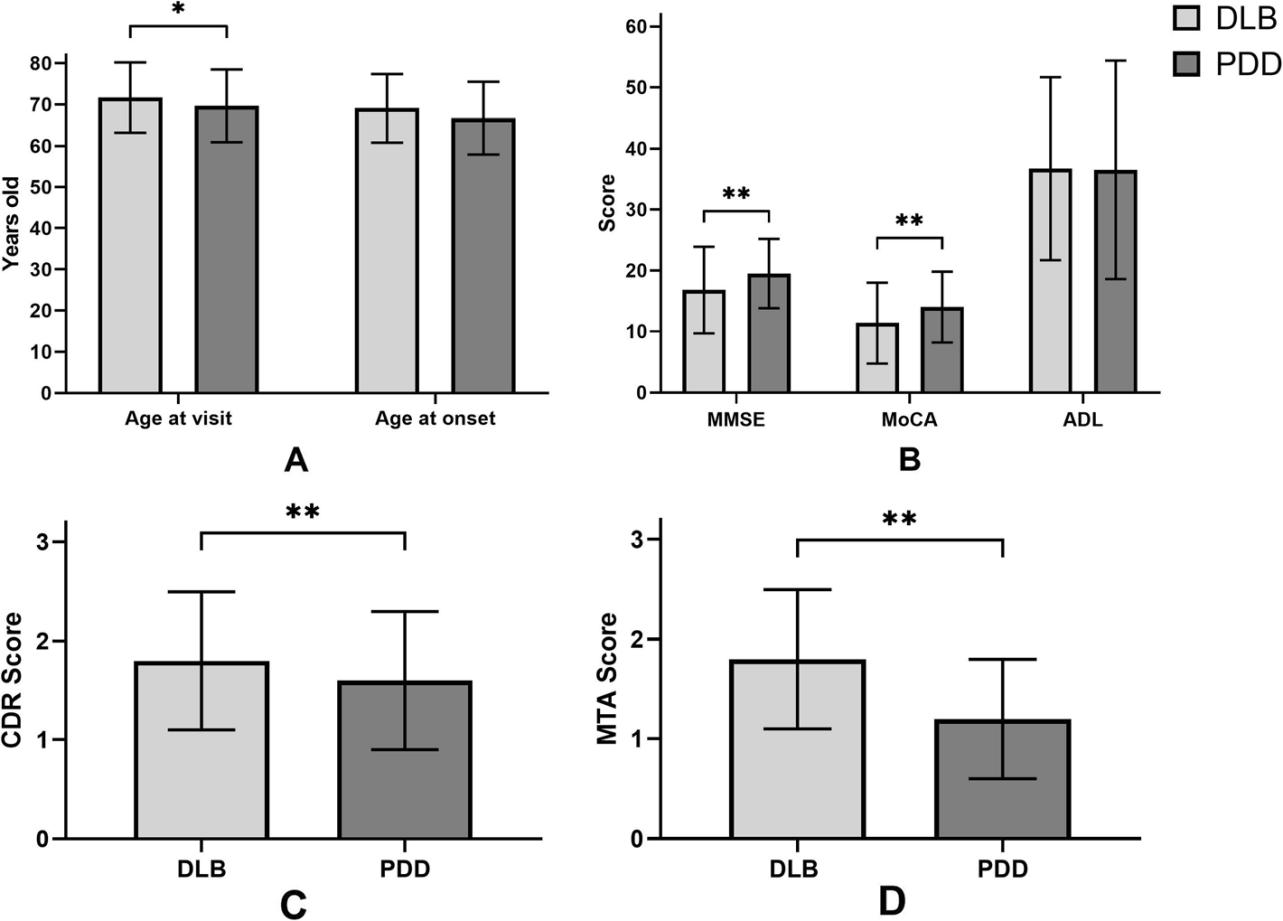
Clinical characteristics: comparison of diagnosis for patients. **a** Comparison of age for DLB and PDD patients. **b** Comparison of clinical evaluations (MMSE, MoCA, ADL) for DLB and PDD patients. **c** Comparison of CDR score for DLB and PDD patients. **d** Comparison of MTA score for DLB and PDD patients. DLB indicates Dementia with Lewy bodies; PDD, Parkinson’s disease dementia; MMSE, Mini Mental State Examination; MoCA, Montreal Cognitive Assessment; ADL, Activities of daily living; CDR, Clinical Dementia Rating; MTA, scale for Medial Temporal lobe Atrophy. *p < 0.05; ** *p* < 0.001

Patients with DLB comprised 5.6% (*n* = 348, 95% CI: 5.0, 6.2) of all dementia cases, while those with PDD comprised 1.7% (*n* = 107, 95% CI: 1.4, 2.0) of all dementia cases. The proportion of those with DLB was higher than that of those with PDD (χ2 = 132.5, *p* < 0.001). Patients with PDD were significantly younger than their DLB counterparts (69.7 ± 8.8 vs. 71.7 ± 8.5, *p* = 0.049), while the difference was not seen in age at onset (shown in Fig. 2a). Patients with DLB had a poorer performance on the MMSE (16.8 ± 7.1 vs. 19.5 ± 5.7, *p* = 0.001), the MoCA (11.4 ± 6.6 vs. 14.0 ± 5.8, *p*<0.001) (shown in Fig. 2b), the CDR (1.8 ± 0.7 vs. 1.6 ± 0.7, *p* = 0.002) (shown in Fig. 2c), and the MTA (1.8 ± 0.7 vs. 1.2 ± 0.6, *p* = 0.002) (shown in Fig. 2d) compared to those with PDD. In other words, patients with DLB demonstrated a poorer cognitive situation at their first visit than patients with PDD.

### LBD in memory clinics

**Fig. 3:**
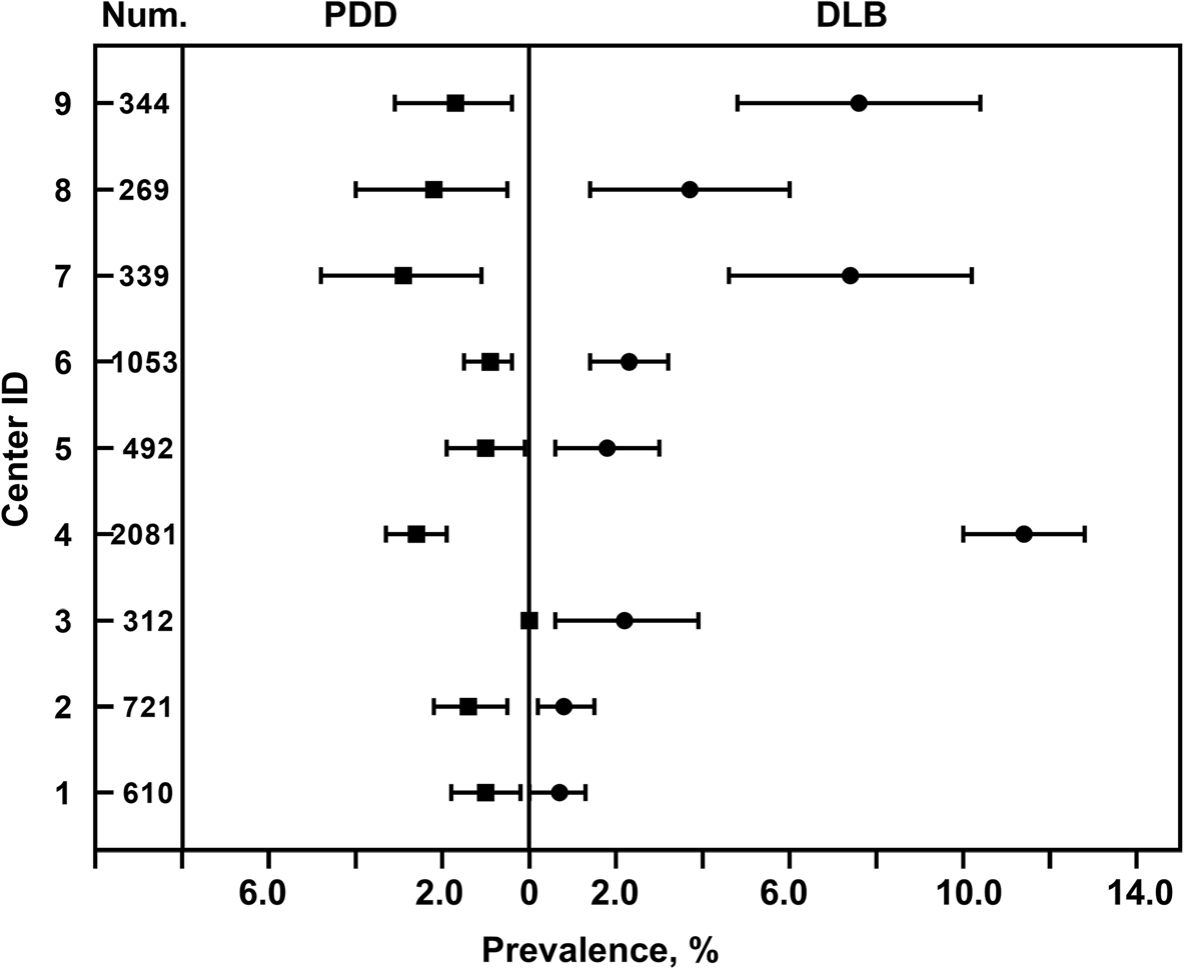
Prevalence of DLB and PDD by center. Intervals represent 95% CI. The total number of dementia subjects for each center are denoted in column Num. DLB, Dementia with Lewy bodies; PDD, Parkinson’s disease dementia

Differences among centers were apparent. Of the nine centers, the prevalence of those with DLB ranged from 0.7 to 11.4% and those with PDD ranged from 0.0 to 2.9%. The prevalence of those with DLB and PDD in all centers and their 95% CI are presented in Fig. 3. The prevalence rates, along with wide CI, suggests that some centers only contributed to a small number of cases while others contributed to a larger proportion of cases. In most memory clinics, the prevalence of those with DLB was higher than that of those PDD. Meanwhile, the prevalence of those with DLB in center 4 showed significant difference from other centers.

### Gender differences in LBD

**Fig. 4:**
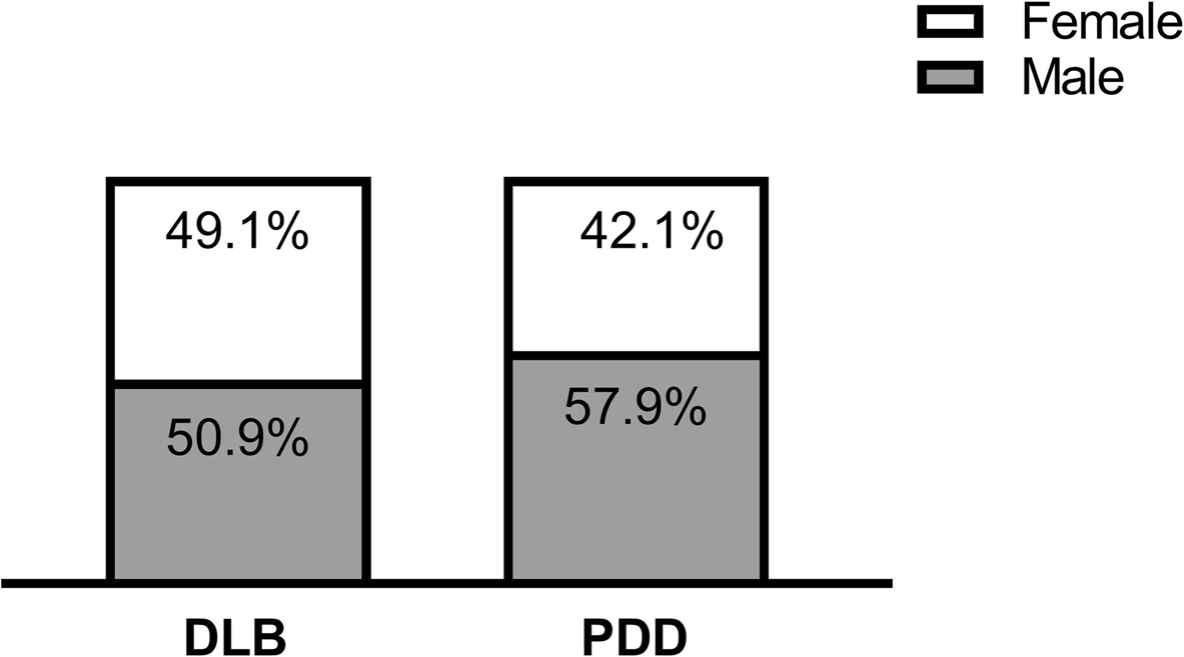
Proportion of male and female patients in DLB/PDD group. DLB indicates Dementia with Lewy bodies; PDD, Parkinson’s disease dementia

In the sampled population, 177 (50.9%) patients with DLB were male and 62 (57.9%) with PPD were male (Fig. 4.) There was no significant difference in gender between the DLB and PDD groups.

An age-stratified analysis of gender in those with DLB and PDD was also conducted (Table 2). For people < 50 years old and 60–69 years old, DLB was a more common diagnosis in females, while the contrary was true in those with PDD. Meanwhile, it was more common of those with PDD who 50–59 years were old to be female. Before the age of 80 years old, the proportion of a DLB diagnosis was increased with age both in males and females, with similar findings in PDD.

**Table 2:** Gender difference in DLB and PDD according to age

	DLB		PDD	
	No.	(%)	No.	(%)
**Age at first consultation with the diagnosis < 50 years**	3	0.9%	1	0.9%
Male	1	0.3%	1	0.9%
Female	2	0.6%	0	0.0%
**Age at first consultation with the diagnosis 50–59, years**	27	7.8%	14	13.1%
Male	14	4.0%	5	4.7%
Female	13	3.8%	9	8.4%
**Age at first consultation with the diagnosis 60–69, years**	105	30.2%	35	32.7%
Male	42	12.1%	25	23.4%**
Female	63	18.1%	10	9.3%
**Age at first consultation with the diagnosis 70–79, years**	153	43.9%	42	39.3%
Male	83	23.8%	22	20.6%
Female	70	20.1%	20	18.7%
**Age at first consultation with the diagnosis 80+, years**	60	17.2%	15	14.0%
Male	37	10.6%	9	8.4%
Female	23	6.6%	6	5.6%

*DLB* Dementia with Lewy bodies, *PDD* Parkinson’s disease dementia

** *p* < 0.001

**Fig. 5:**
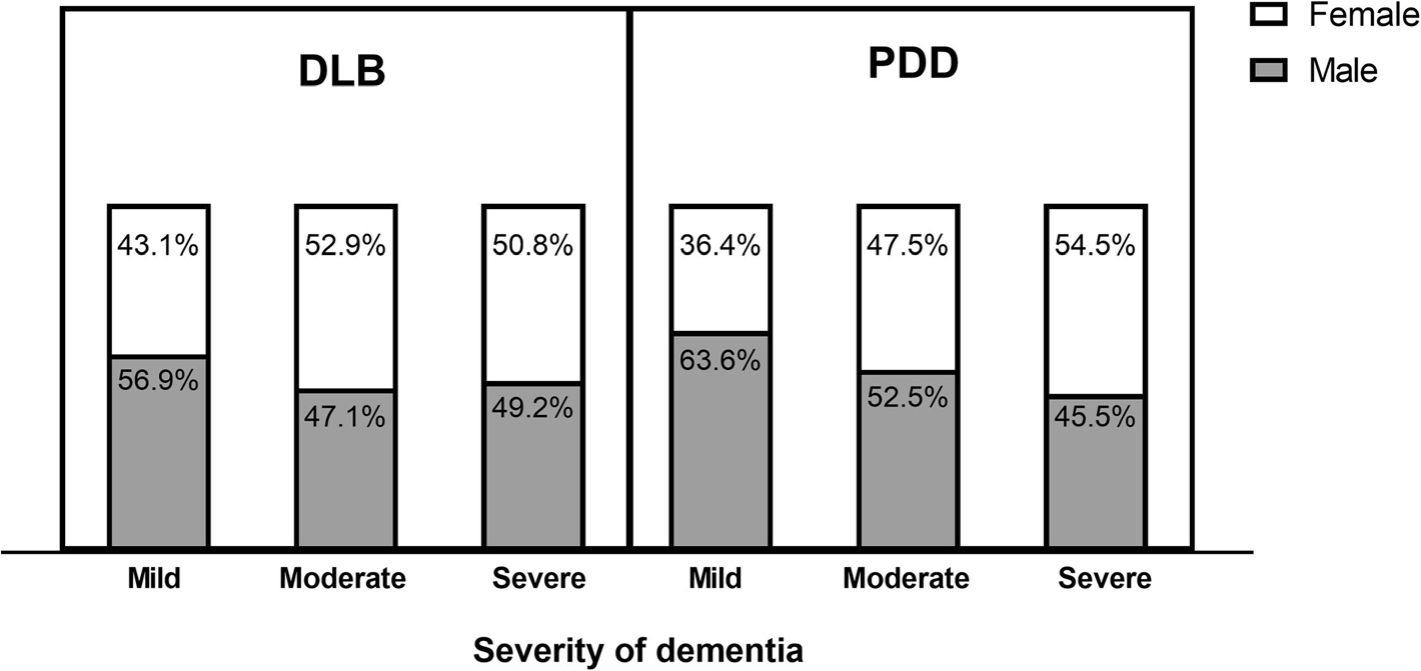
Gender difference in DLB and PDD according to severity. DLB indicates Dementia with Lewy bodies; PDD, Parkinson’s disease dementia

The comparison of gender by severity of dementia at the first visit to the memory clinics is displayed in Fig. 5. Females, both with DLB and PDD, were more likely to attend a memory clinic when their symptoms were severe (50.8% with DLB, 54.5% with PDD). Males, on the other hand, preferred to visit when their symptoms were mild (56.9% with DLB, 63.6% with PDD). Females with either DLB or PDD were more severe at their first visit, compared to males.

## Discussion

In recent years, the clinical prevalence of LBD was infrequently reported in the larger population. Clinical DLB accounts for 5.6% (5.0–6.2%) of all dementia cases, which is slightly higher than the UK memory services proportion, with 4.6% (95% CI: 4.0, 5.2) [2], as well as clinical samples derived from systematic review with 4.2% [25], while it was lower when compared with Turkey (9.7%) or other regions [26, 27]. In this cross-sectional, population-based study, a prevalence of those with DLB was 1.05% in individuals aged 60 years or older and the overall prevalence of dementia was 10.36%, which was much higher than previously reported in nine memory clinics [28]. The clinical prevalence of DLB in the current study, accounting for 5.4% (4.9–5.9%), was strikingly higher than previous research in China and Japan [29, 30]. In Chan’s study using patients in a psychogeriatric unit, the overall prevalence of DLB was 2.9% [31]. This suggests a relatively low prevalence of DLB in the Chinese population.

It is worth noting that the prevalence in the current study was diagnosed using the DSM-IV and the 2005 third report of the DLB consortium; the prevalence at current recommended criteria is likely to be higher. With the update of the DSM-5 and the fourth consensus criteria, the improvement of sensitivity and accuracy makes it easier to identify probable DLB when making a direct comparison to the 2005 and 2017 criteria for DLB [32].

### Gender distribution

A primary finding of the current study was the slight predominance of males in the LBD population, with the proportion of 52.5% (*n* = 239), which is consistent with a previous study [33]. The gender distribution in those with DLB and PDD both showed the predominance of males (50.9% in those with DLB, 57.9% in those with PDD), with no differences in females. Because of the poor understanding and overlap of clinical features between DLB and PDD, gender distribution for LBD varied. As shown in a cross-sectional study with LBD, women were more common in those diagnosed with DLB, but not in those diagnosed with PDD [34], while several anatomopathological studies have shown a male predominance in DLB. Other scientific literature on gender distribution in dementia traditionally reports a more pronounced prevalence of men in those with PD (2:1 male/female ratio) [35] and PDD (61.3% in Sweden) [36],, as well as in those with DLB (62.6% in Sweden [36]; 51.7% in our previous study [28]). Gender differences have also been reported in the initial symptoms of DLB. Utsumi et al. [37] suggested considering DLB in elderly aged men who report RBD, because males tend to present with more RBD symptom than females. Therefore, the gender distribution of LBD may have a greater disparity, with males at a higher risk.

The age-stratified analysis of gender in LBD was also conducted in the current study. Most LBD patients were in the age group of 70–79 years, followed by 60–69 years at their first visit. In Rait’s study, people 60–79 years old had a higher incidence of LBD compared with others [38], which is younger than in the current study (the most common age reported here is 70–79 years). For those aged 60–69 years, DLB is more common in females, but is more balanced in age groups younger than 60 years. By contrast, for people older than 70 years old, more males had DLB than females. The mean age of PDD (69.7 ± 8.8 years) in the current study was younger than in many other studies [6, 39], demonstrating a preference of males that increased with age from 60 years old. As reported, being male was a risk factor for LBD [40], especially in older adults.

A severity-stratified analysis of gender in LBD is presented in Fig. 5. Males were more likely to visit when symptoms were mild, whether presenting with DLB (56.9%, *n* = 66) or with PDD (63.6%, *n* = 35), while females with DLB mostly visited at moderate and serve symptom levels, mostly in balance with males. The proportion of females with PDD increased with severity and overtook the male proportion at severe cases. Therefore, more attention to females at early stages should be paid, particularly those with a high risk of LBD.

### Clinical characteristics at first diagnosis

DLB and PDD were classified by means of the one-year rule [18], showing that patients with a diagnosis of PDD (mean visit age = 69.7 ± 8.8) were younger compared with DLB (mean visit age = 71.7 ± 8.5) at their first visit to a memory clinic (*p* = 0.049), possibly related to their onset age or progression. Onset age of those with PDD (66.7 ± 8.8) was, indeed, younger than those with DLB (69.7 ± 8.8) in the current study. In a previous study, data regarding the comparative age related to the prevalence of PDD and DLB was limited, with some suggesting that DLB patients may be younger at symptom onset than those with PDD, with more hallucinations and cognitive fluctuations, while others reported younger age at disease onset in PDD or no real differences between the disorders [41]. The diagnostic criteria for PDD [19] require impairment in two cognitive domains plus impairments in the ADL scale because of CI, which developed within the context of established PD. Patients with PD were younger (less than 65 years old) [42] at onset than patients with DLB. Patients with PD have more opportunity to know dementia with PD, perhaps explaining why PDD patients visit a memory clinic earlier than patients with DLB. Because the age of onset of memory impairment in patients with PDD was not recorded and analyzed in the current study, it is hard to further clarify the age of memory impairment in those with PDD and to subsequently study the progress of memory impairment.

Those with PDD demonstrated better cognition than those with DLB at the time of dementia diagnosis in the current study, which is reflected in the higher MMSE and MoCA scores, as well as the lower average degree of CDR than DLB patients. Previous research on cognition in LBD was also inconsistent. In Kramberger et. al’s cohort study, the average MMSE score in the PDD group (*n* = 198, mean score = 21.2 ± 5.5) was balanced with the DLB group (*n* = 835, mean score = 21.3 ± 4.9). Contrarily, the Swedish Dementia Registry data showed that the PDD group (*n* = 764) had a significantly lower MMSE score (20.7 vs. 21.4, *p* = 0.001) than the DLB group (*n* = 1110).

In the current study, either a CT or structural MRI was used to detect regional brain atrophy. A visual assessment of MTA, semi-quantified by a five-grade system (MTA score), has been previously used in AD [43], and is comparable in accuracy to a volumetric analysis [44]. There were no significant differences in age or sex ratio in the two groups. Previous autopsy studies have demonstrated that white matter hyperintensities (WMH), cerebral amyloid angiopathy (CAA), and medial temporal lobe atrophy were common in dementia and the rate of MTA was useful as a biomarker for predicting the degree of AD-related pathology in LBD [45]. Those with DLB (MTA score = 1.8 ± 0.7) presented with a more serve MTA than those with PDD (MTA score = 1.2 ± 0.6) (shown in Fig. 2), consistent with Joki et al’s research [46]. This may explain the better cognitive performance in those with PDD compared to those with DLB. AD patients showed the most severe atrophy of the medial temporal lobe (MTA score = 2.24 ± 0.95), followed by DLB patients (1.81 ± 1.04), PDD patients (1.43 ± 1.05), PD without dementia patients (MTA score = 0.65 ± 0.76), and normal subjects (0.44 ± 0.50), in that order. Somewhat differently, the age in the current study was younger than in Joki et al’s research.

### Region distribution

Various centers differed in the proportion of patients with DLB and PDD. Among the nine centers encountering seven or more DLB cases and 0–54 PDD cases, informant-endorsed DLB ranged from as low as 0.7% (center 1) to as high as 11.4% (center 4) (shown in Fig. 3), while PDD ranged from 0% (center 3) to 2.9% (center 7).

Diagnostic variability of LBD can be interpreted in multiple different ways. It is possible that LBD patients naturally occurred at lower proportions at certain centers, thereby reflected in the informant reports. With the development of molecular imaging technology, PET and SPECT were applied in the diagnosis. DAT-scans can confirm the loss of the dopaminergic transporters; severe nigrostriatal dopaminergic degeneration occurs in DLB, but not in AD or most other dementia subtypes. The sensitivity and specificity of ^123^I-FP-CIT SPECT scans to clinically diagnose probable DLB are 77.7 and 90.4%, respectively. In patients with dementia and no history of Parkinson’s disease, abnormal scanning can prompt a diagnosis of DLB [47].

For the diagnosis and treatment of dementia, caregiver ratings are important. Caregivers’ recall bias, particularly for the time and frequency of symptoms, may contribute to a difference in the diagnosis of LBD. Situations such as caregivers not sleeping in the same bedroom as the patients may become particularly problematic in leading to inaccuracies in the description of the RBD.

Behavioral and psychological symptoms (BPSD) are reported to be common among those with DLB, PDD, and AD. This overlap of neuropathology and symptoms (behavioral, psychological, and cognitive) often makes an accurate diagnosis difficult. Hallucinations in AD can occur in any sensory modality, but visual hallucinations most commonly occur in AD and the rate of BPSD is dependent on AD stage, with low rates of psychosis in the prodromal and early AD stages and higher rates in middle and later stages [48]. Therefore, there are some difficulties in the diagnosis of visual hallucinations. Those with PDD and DLB were relatively more impaired and declined more rapidly than those with AD in visuospatial ability, but did not differ from each other (DLB ≈ PDD < AD).

Of consequence to the current study, a robust finding was, indeed, that various centers differed in the awareness and diagnosis of LBD. The proportion of those with DLB ranged from 0.7 to 11.4%, while the proportion of those with PDD ranged from 0.0 to 2.9%, which is significantly lower than previous reports. LBD comprises 15–20% of cases of dementia in pathological studies [6]. However, clinically, the prevalence is much lower, with DLB prevalence reported to be 4.2–5% [49] of all patients with dementia and PDD prevalence reported to be 3.6% [8]. Molecular imaging was used to clarify atypical LBD patients in one of the memory clinics (center 4). Chinese clinical features of DLB were addressed, with ^11^C-PiB PET and ^18^F-FDG PET scans confirming Aβ deposition and revealing hypometabolism in the cortex. The clearance rate of radioactivity was slower, symmetrically or asymmetrically, in the cortex of the frontal lobe, parietal lobe, lateral temporal lobe, precuneus, posterior cingulate, and occipital lobe in DLB patients. Molecular imaging allowing for in vivo detection of fibrillar plaques, hypometabolism, and Tau, as well as ^123^I-FP-CIT SPECT, is currently considered as the gold standard for the diagnosis of DLB [50]. Validated diagnostic methods for LBD could improve the accuracy of diagnoses, particularly molecular imaging, but are not generally available for clinical and population-based studies because of their high costs [51].

The importance of correctly diagnosing DLB has been highlighted in a previous review [1], which identified the far-reaching consequences of having LBD that may not be appreciated without a diagnosis. The gravest danger of a lack of diagnosis is the inadvertent use of anti-psychotics, which can be fatal in patients with DLB if neuroleptic malignant syndrome is triggered and commonly leads to the worsening of their debilitating movement disorder.

Strengths of the current study include being the first in China, utilizing a large sample size, and being a multicenter study. The current study provides reference for a clinical diagnosis of LBD. Potential limitations include the use of a retrospective design, which could introduce recall bias; however, to limit this bias, the data was collected from contemporaneously written medical records rather than being based on recall by patients or clinicians. Additionally, only records from voluntary memory clinics were collected, rather than national neurology, geriatric, psychiatry, or movement disorder clinics, which dementia and movement disorder patients also visit. Another potential limitation is that the diagnoses of the patients were not subsequently validated by autopsy, which is the gold standard for a diagnosis. Finally, not all the patients were diagnosed using the updated protocols. This results in data bias, further indicated by the inconsistencies between clinic and population prevalence rates. Nevertheless, all case report forms were verified by an expert panel that assessed each case with reference to the relevant diagnostic criteria.

## Conclusion

LBD comprised 5.4% (4.9–5.9%) of the samples and 7.3% (6.7–8.0%) of all dementia types. Patients with DLB comprised 5.6% (95% CI: 5.0, 6.2), while those with PDD comprised 1.7% (95% CI: 1.4, 2.0) of all types of dementia. Variations of diagnoses exist in different regions and the true clinical prevalence of LBD is likely to be underestimated in China and other regions.

## Data Availability

The datasets used and/or analyzed during the current study are available from the corresponding author on reasonable request.
